# Photosynthetic capacity, leaf respiration and growth in two papaya (*Carica papaya*) genotypes with different leaf chlorophyll concentrations

**DOI:** 10.1093/aobpla/plz013

**Published:** 2019-03-08

**Authors:** Jéssica Sousa Paixão, Jefferson Rangel Da Silva, Katherine Fraga Ruas, Weverton Pereira Rodrigues, José Altino Machado Filho, Wallace de Paula Bernado, Deivisson Pelegrino Abreu, Luciene Souza Ferreira, Julian Cuevas Gonzalez, Kevin Lee Griffin, José Cochicho Ramalho, Eliemar Campostrini

**Affiliations:** 1 Setor de Fisiologia Vegetal, Laboratório de Melhoramento Genético Vegetal, Centro de Ciências e Tecnologias Agropecuárias, Universidade Estadual do Norte Fluminense, Avenida Alberto Lamego, Parque Califórnia, Campos dos Goytacazes, Rio de Janeiro, Brazil; 2 Centro de Citricultura Sylvio Moreira, Instituto Agronômico, Rodovia Anhanguera, Cordeirópolis, São Paulo, Brazil; 3 Instituto Capixaba de Pesquisa, Assistência Técnica e Extensão Rual, Rua Afonso Sarlo, Bento, Ferreira, Vitória, Espírito Santo, Brazil; 4 Agronomy Department, University of Almería, ceiA3, Ctra. Sacramento s/n, Almería, Spain; 5 Department of Earth and Environmental Sciences, Columbia University, Lamont-Doherty Earth Observatory, Palisades, NY, USA; 6 Department of Ecology, Evolution, and Environmental Biology, Columbia University, New York, NY, USA; 7 Lab. Interações Planta-Ambiente & Biodiversidade (PlantStress&Biodiversity), Linking Landscape, Environment, Agriculture and Food (LEAF), Departamento de Recursos Naturais, Ambiente e Território (DRAT), Instituto Superior de Agronomia (ISA), Universidade de Lisboa (ULisboa), Av. República, Oeiras, Portugal; 8 GeoBioTec, Faculdade de Ciências Tecnologia, Universidade NOVA de Lisboa, Caparica, Portugal

**Keywords:** Chlorophyll fluorescence, leaf respiration rates, net photosynthesis, nitrogen concentration, RuBisCO oxygenation/carboxylation rates

## Abstract

Golden genotype of papaya (*Carica papaya*), named for its yellowish leaves, produces fruits very much appreciated by consumers worldwide. However, its growth and yield are considerably lower than those of other genotypes, such as ‘Sunrise Solo’, which has intensely green leaves. We undertook an investigation with the goal of evaluating key physiological traits that can affect biomass accumulation of both Golden and Sunrise Solo genotypes. Papaya seeds from two different genotypes with contrasting leaf colour ‘Sunrise Solo’ and Golden were grown in greenhouse conditions. Plant growth (plant height, leaf number, stem diameter, leaf area, plant dry weight), leaf gas exchanges, leaf carbon balance, RuBisCO oxygenation and carboxylation rates, nitrogen, as well as chlorophyll concentrations and fluorescence variables were assessed. Although no significant differences were observed for photosynthetic rates between genotypes, the accumulation of small differences in photosynthesis, day after day, over a long period, might contribute to some extend to a higher C-budget in Sunrise Solo, higher leaf area and, thus, to higher productivity. Additionally, we consider that physiological processes other than photosynthesis and leaf respiration can be as well involved in lower growth and yield of Golden. One of these aspects could be related to the higher rates of photorespiration observed in Sunrise Solo, which could improve the rate of N assimilation into organic compounds, such as amino acids, thus contributing to the higher biomass production in Sunrise Solo relative to Golden. Further experiments to evaluate the effects of N metabolism on physiology and growth of Golden are required as it has the potential to limit its yield.

## Teaser

This study provides the first complete picture of leaf carbon balance in two economically important genotypes of papaya and demonstrates that neither stomatal effects nor reduced photochemical and carboxylation capacities of the Golden genotype affected CO_2_ assimilation through photosynthesis. We consider that physiological processes other than photosynthesis/leaf respiration (LCB) can be as well involved in the worst growth and yield of Golden. For example, higher rates of photorespiration were observed in Sunrise Solo, which could improve the rate of N assimilation into organic compounds, thus contributing to the higher biomass production in Sunrise Solo relative to Golden.

## Introduction

Papaya is the most economically important species within the Caricaceae family and it is widely cultivated not only for fruit consumption, but also for the proteolytic enzyme papain, which has several commercial and medical uses (Villegas 1997; Campostrini and Glenn 2007; Carr 2014). In 2016, there were an estimated 441 964 ha of papaya cultivated worldwide, with an annual production of >13 million t of fresh fruit. The principal producers were India (5.69 million t from 133 000 ha), Brazil (1.42 million t from 30 372 ha), Mexico (0.95 million t from 16 820 ha) and Indonesia (0.90 million t from 9980 ha) (FAOSTAT 2016). Although Golden fruits are better accepted by consumers worldwide due to the superior appearance of the fruits, greater transport resilience and longer post-harvest, Golden plants are less vigorous (Torres-Netto *et al.* 2009; Castro *et al.* 2014) and lower yielding compared to ‘Sunrise Solo’ (90 vs. 120 t ha^−1^ in the whole cycle) (Caliman Agrícola, Linhares, Espiríto Santo, Brazil, pers. comm.; Costa and Pacova 2003). Lower growth vigour and yield were first assumed to be related to the reduced chlorophyll and nitrogen (N) content of the leaves (Torres-Netto *et al.* 2009; Castro *et al.* 2014).

A substantial fraction of leaf N is allocated to the photosynthetic apparatus and invested in chlorophyll, chlorophyll-binding proteins and, especially, RuBisCO (Griffin and Seemann 1996; Zhang *et al.* 2008; Sage 2013), thus higher N content promotes better photosynthetic performance (Hikosaka and Terashima 1995), and plays a crucial role in the plant ability to trigger acclimation mechanisms (Ramalho *et al.* 1999; Carelli *et al.* 2006). RuBisCO alone can account for up to 50 % of leaf soluble protein (Spreitzer and Salvucci 2002) and for 20–30 % of total leaf nitrogen (Makino 2003). Thus, the low leaf N concentration of Golden leaves could negatively affect their photosynthetic capacity and therefore, growth and yield (Eckardt 2009; Akram and Ashraf 2011). However, a previous study showed that Golden had similar CO_2_ uptake rates to ‘Sunrise Solo’ (Castro *et al.* 2014), which suggests that physiological processes other than photosynthesis are likely responsible for the reduced growth and yield of Golden. Previous work on leaf pigments demonstrated that similar rates of canopy photosynthesis can be maintained in soybean with dramatically lower leaf chlorophyll and 9 % lower leaf nitrogen (Walker *et al.* 2017). Additionally, Slattery *et al.* (2017) reported that chlorophyll content can be drastically reduced with little impact to canopy photosynthesis, suggesting an over-investment in chlorophyll and an under-utilization of photosynthetic biochemical capacity in modern soybean cultivars. Slattery *et al.* (2017) further showed that chlorophyll deficiency led to greater rates of leaf-level photosynthesis per absorbed photon early in the growing season when mutant chlorophyll content was *ca.* 35 % of the wild-type, but there was no effect on photosynthesis later in the season when mutant leaf chlorophyll approached 50 % of the wild-type. Despite a >50 % chlorophyll reduction, there was little negative impact on both biomass accumulation and yield. The small negative effects that were present were likely due to a pleiotropic effect of the mutation that was linked to lower water use efficiency (WUE) that may have dampened any photosynthetic benefits of reduced chlorophyll content, especially since significant drought conditions were experienced during the work (Slattery *et al.* 2017).

Other authors have studied the relationship between photosynthetic pigment content and photosynthetic capacity in papaya plants. Castro *et al.* (2014) showed that the reduction in the maximum quantum yield of primary photochemistry (*F*_*v*_*/F*_*m*_) in Golden was only observed when total chlorophyll concentration was <400 µmol m^−2^, while in ‘Sunrise Solo’ the *F*_*v*_*/F*_*m*_ reduction was observed when total chlorophyll concentration was <600 µmol m^−2^. This lack of effect of total chlorophyll concentration on *F*_*v*_*/F*_*m*_ ratio may indicate that there was a reduction in the generation of reactive oxygen species from over-excited reaction centres (Vass and Cser 2009). Thus, by reducing total light energy absorption (<*total chlorophyll concentration*) and conserving antioxidant capacity, light stress resilience may be improved (Foyer and Shigeoka 2011). Despite the relevance of these previous studies, a full analysis of the relationships between chlorophyll concentration, gas exchange, leaf respiration and leaf carbon balance (LCB) was not performed.

Slattery *et al.* (2017) hypothesized that decreasing leaf absorbance through reduced chlorophyll content could improve light penetration into a crop canopy, so that sun leaves would reduce over-saturation at midday, while allowing more light to reach the lower canopy. This would stimulate maximum net photosynthetic rates (*A*_net_) in shade leaves, thereby potentially improving canopy photosynthesis, and potential yield. However, papaya plants exhibit spiralled phyllotaxis in a 3:8 configuration (Campostrini *et al.* 2018) which might impact canopy light absorption. There are three leaves positioned clockwise or counterclockwise within each 360° turn around the trunk, and spiral leaf insertion on the trunk. This leaf arrangement is associated with long petioles and allows for efficient light distribution in the papaya canopy (Ferraz *et al.* 2016). Thus, the hypothesis proposed by Slattery *et al.* (2017) might be less relevant for papaya. However, considering that photosynthetic performance is maintained for lower leaf chlorophyll and N contents, it might be envisaged a somewhat decreased application of N to reduce costs, leaching and soil contamination (Neeteson 1995).

Net leaf carbon gain estimated from leaf photosynthesis alone typically leads to an overestimation of plant performance (Escalona *et al.* 2012). Therefore, leaf respiration in the dark and in the light must also be considered to obtain an accurate C-balance estimate. Indeed, leaf respiration occurs continuously and even small changes in this process can result in substantial variation in the plant C-balance and, hence, in growth and yield (Poni *et al.* 2009; Flexas *et al.* 2010; Griffin and Heskel 2013; Tomás *et al.* 2014). Moreover, leaf respiration is positively correlated to leaf N concentration due to the links between the tricarboxylic acid pathway and N metabolism (Crous *et al.* 2012). In this context, Golden and Sunrise Solo could have different leaf respiration rates in response to the contrasting leaf N contents present in the leaves of each genotype (Castro *et al.* 2014), and could contribute to explain the observed differences in growth and yield between these cultivars. Therefore, it will be important to quantify respiration rates both in the dark (*R*_dark_) and in the light (*R*_light_) to better understand LCB.

High rates of photorespiration in C3 plants such as papaya, supports the efficient uptake of transiently available nitrogen, increasing the N assimilation into organic compounds (Busch *et al.* 2018). Moreover, in addition to its relevance to N metabolism, photorespiration consumes high-energy reductant (four electrons per oxygenation reaction), and thus plays an important role in dissipating excess energy. This serves to mitigate photoinhibition in high light, and may be crucial to the maintenance of high photosynthetic rates, and a positive C-balance (Wingler *et al.* 2000; Hochberg *et al.* 2013). Therefore, changes in photorespiration rates of papaya genotypes must also be taken into account to better understand the influence of this important physiological process on plant growth.

In Brazil, the two most important papaya genotypes are ‘Sunrise Solo’ and Golden, both from Solo group (Trindade *et al.* 2000). To the best of our knowledge, this is the first work designed to analyse LCB (photosynthesis, respiration and photorespiration), in different papaya genotypes. Previous study focused on photosynthetic rates and maximum quantum yield of primary photochemistry failed to explain the differences in plant growth of Golden and Sunrise Solo genotypes (Castro *et al.* 2014). Thus, this work is a step forward, aiming at understand the implications of C-balance in papaya growth and yield. In fact, given the economic importance of papaya crop (Campostrini and Glenn 2007; Campostrini *et al.* 2018), it is imperative to better understand physiological underpinnings of its growth and production. Thus, we undertook an investigation with the goal of evaluating whether photorespiration, photosynthetic capacity, leaf respiration and, therefore, LCB can affect the growth of papaya plants with contrasting leaf chlorophyll contents. We hypothesize that the Golden genotype has higher leaf respiration rates in both light and dark, negatively impacting LCB, and affecting the growth of plants.

## Materials and Methods

### Plant material and growth conditions

This study was conducted in a greenhouse at the Universidade Estadual do Norte Fluminense, located in Campos dos Goytacazes, Rio de Janeiro (21°44′47″S; 41°18′24″W), with natural fluctuations of light, temperature and relative humidity **[see****Supporting Information—Fig. S1****]**. The 147 m^2^ greenhouse was covered with both 150 µm thick plastic and shading screen, transmitting 60 % of the solar radiation. The east-west oriented structure was closed on all sides with anti-aphid screen. The study was carried out from January to March 2017 (summer season).

**Figure 1.**
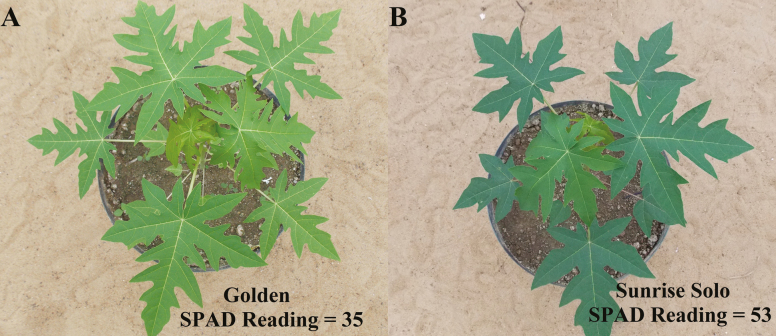
Golden (A) and Sunrise Solo (B) plants used in the experiment.

Papaya seeds from two different genotypes with contrasting leaf colour [‘Sunrise Solo’ (intensely green leaves) and Golden (yellowish-green leaves); Fig. 1] were sown in 0.28 L pots inside the greenhouse. Forty-one days after sowing, nine plants of each genotype were transferred to 40 L high-density polyethylene pots (1 plant per pot). Pots were wrapped in a reflective aluminized blanket to avoid soil over-heating. The substrate consisted of soil [Dystrophic Yellow Latosol (Embrapa 2006)], sand and cattle manure (2:1:1, v/v). Fertilization was made during substrate preparation, with a total of 60 g per pot of CaO; 10.2 g per pot of N (NH_4_^+^:NO_3_^−^ ratio = 1.5); 4.5 g per pot of P_2_O_5_; 6.6 g per pot of K_2_O; 0.39 g per pot of Mg; 1.8 g per pot of S; 0.015 g per pot of Cu; 0.138 g per pot of Fe; 0.018 g per pot of Mn; 0.006 g per pot of Mo and 2 g per pot of Fritted Trace Elements (FTE Br-12, Nutriplant Indústria e Comércio S/A, Brazil). All pots were fully irrigated every 2 days to maintain them under field capacity, throughout the entire experiment.

Air temperature and relative humidity were recorded using a data logger (WatchDog Model 450, Spectrum Technologies, Plainfield, IL, USA). Photosynthetic photon flux density (PPFD) was monitored from 25 days after transferring plants to the 40 L pots onward, with a quantum light sensor (model LightScout, Spectrum Technologies, Plainfield, IL, USA) and recorded (WatchDog Model 450, Spectrum Technologies, Plainfield, IL, USA). Air vapour pressure deficit (VPD) was calculated according to Jones (1992). Climate variables are shown in **Supporting Information—Fig. S1**. Mean values of maximum, average and minimum air temperatures throughout the experiment were 31.71, 26.34 and 21.24 °C, respectively. Mean values of maximum, average and minimum relative humidity throughout the experiment were 89.3, 74.1 and 49.10 %, respectively. Mean values of maximum, average and minimum VPD throughout the experiment were 3.08, 1.13 and 0.28 kPa, respectively. Finally, mean values of maximum, average and minimum PPFD throughout the experiment were 613, 260 and 3 µmol m^−2^ s^−1^, respectively.

### Plant growth

Plant height measured with a ruler, leaf number, and stem diameter measured with a digital calliper (±0.01 mm precision) were determined twice a week, from 15 to 50 days after transferring plants to the 40 L pots, when the length of the central vein of the youngest leaf of each plant was measured. Then, the leaf was tagged so central vein measurements could be taken on the same days as all other morphological measurements through the duration of the experiment. The central leaf vein data were used to calculate the total leaf area of the plants (m^2^) throughout the experiment according to the equation proposed by Posse *et al.* (2009).

### Leaf gas exchanges

Leaf gas exchange measurements were made when the central leaf vein measurements reached stable values (at 43 and 44 days after transferring plants, 5–6th leaf below the plant apex). Light response curves of net photosynthetic rate (*A*_net_) using a Li-Cor 6400XT portable photosynthesis infrared gas analyzer (IRGA) system (Li-Cor Inc., Lincoln, NE, USA) were obtained at 0800, 1200 and 1600 h and made as described in Shapiro *et al.* (2004) taking into consideration the precautions of Pons and Welschen (2002). The system incorporated a CO_2_ controller which was used to set the CO_2_ concentration inside the leaf cuvette to 400 µL L^−1^. The 6 cm^2^ cuvette was fitted with a red–blue light source (6400-02B). The net photosynthetic rate (*A*_net_), stomatal conductance (*g*_*s*_) and transpiration rate (*E*) were determined from the light response curves with 24 levels of PPFD: 1500, 1200, 800, 500, 200, 100, 90, 80, 70 µmol m^−2^ s^−1^ and every 5 units between 70 and 0 μmol m^−2^ s^−1^. The light compensation point (*I*_*c*_, PPFD at which *A*_net_ = 0), the incident quantum yield (Φ_*I*_, slope of the linear portion of the light response curve, between 0 and 300 μmol m^−2^ s^−1^ of PPFD) and maximum photosynthesis (*A*_max_) were obtained from the light response curves.

The rate of respiration in the light (*R*_light_) was estimated using the method originally described in Kok (1948), as the *y*-axis intercept of a first-order linear regression fitted to *A*_net_-irradiance plots to measurements made over the 25–65 µmol m^−2^ s^−1^ irradiance range. All gas exchange data were corrected for the increase in intercellular CO_2_ concentrations (*C*_*i*_) with decreasing irradiance, which can result in reduced rates of photorespiration and increased rates of carboxylation (Villar *et al.* 1994). The correction was applied by adjusting the *R*_light_ through iteration to minimize the intercept of photosynthetic electron transport (*J*) as a function of irradiance (Kirschbaum and Farquhar 1987). *J* was calculated according to Farquhar and von Caemmerer (1982):

$$\begin{array}{l} J=\;\frac{\left[(4(A_{net}+\;R_{light}))(C_{i}+2\Gamma^{*})\right]}{\left(C_{i}-\;\Gamma_{*}\right)} \end{array}$$ (1)

where Γ* is the CO_2_ compensation point in the absence of *R*_light_ (von Caemmerer and Farquhar 1981; 38.6 at 25 °C). The rates of oxygenation and carboxylation by RuBisCO (*V*_*o*_ and *V*_*c*_, respectively) were calculated at intensities of 0 to 1500 μmol m^−2^ s^−1^, according to Farquhar and von Caemmerer (1982):

$$\begin{array}{l} V_{c}=\;\frac{1}{3}\left[\left(\frac{J}{4}\right)+2(A_{net}+R_{light})\right]\; \end{array}$$ (2)

$$\begin{array}{l} V_{o}=\;\frac{2}{3}\left[\left(\frac{J}{4}\right)-(A_{net}+R_{light})\right]\; \end{array}$$ (3)

The effects of varying atmospheric [O_2_] or [CO_2_] on oxygenation (*V*_*o*_) and carboxylation (*V*_*c*_) at each intensity of light used were also calculated according to Farquhar and von Caemmerer (1982):

$$\begin{array}{l} V_{c}=\;\frac{[CO_{2}]V_{c\max}}{[CO_{2}]+K_{c}\;\left(1+\frac{\left[O_{2}\right]}{K_{o}}\right)} \end{array}$$ (4)

and

$$\begin{array}{l} V_{o}=\;\frac{[O_{2}]V_{o\max}}{[O_{2}]+K_{o\;}\left(1+\frac{\left[CO_{2}\right]}{K_{c}}\right)} \end{array}$$ (5)

Equations (4) and (5) used the RuBisCO kinetic constants (*K*_*c*_ = 404.9 μmol mol^−11^; *K*_*o*_ = 278.4 mmol mol^−1^) previously determined by Bernacchi *et al.* (2001) at 25 °C, and calculated *V*_*c* max_ as:

$$\begin{array}{l} V_{c\max}=\;\frac{A_{net}-R_{light}}{\frac{[CO_{2}]-\Gamma^{*}}{[CO_{2}]+K_{c}\left(1+\frac{\left[O_{2}\right]}{K_{c}}\right)}} \end{array}$$ (6)

Γ* (the CO_2_ compensation point in the absence of *R*_light_) depends on the RuBisCO specificity factor, O_2_ partial pressure and is calculated according to von Caemmerer and Farquhar (1981) for real leaf temperature:

$$\begin{array}{l} \Gamma^{*}=\;\frac{0.5V_{o\max}K_{c}[O_{2}]}{V_{c\max}K_{o}}\; \end{array}$$ (7)

Here we use the specificity presented in Cousins *et al.* (2010), the ambient O_2_ concentration and the temperature response function of Brooks and Farquhar (1985) to determine Γ*. The *V*_*o*_, *V*_*c*_ and *V*_*c*_:*V*_*o*_ ratio values reported here were calculated at ambient CO_2_ concentration.

In order to obtain the rate of respiration in the dark (*R*_dark_), the value of *A*_net_ at 0 μmol m^−2^ s^−1^ of PPFD was determined at 2000 h. The *A*_net_, *g*_*s*_ and *E* values were used to calculate the intrinsic WUE (iWUE) and instantaneous WUE as the slope of the linear relationship between *A*_net_ and *g*_*s*_ or *A*_net_ and *E*, respectively, at 200, 500, 800, 1200 and 1500 μmol m^−2^ s^−1^ of PPFD. In addition, LCB for the experiment was estimated using the following equation:

$$\begin{array}{l} LCB\;=\frac{A_{net\;0800\;h}+A_{net\;1200\;h}+\;A_{net\;1600\;h}}{R_{dark}+\;R_{light\;0800\;h\;}+\;R_{light\;1600\;h\;}+\;R_{light\;1200\;h\;}} \end{array}$$ (8)

where *A*_net 0800 h_; *A*_net 1200 h_; *A*_net 1600 h_ are *A*_net_ measured at 0800, 1200 and 1600 h, respectively, and at either 1200 or 1500 μmol m^−2^ s^−1^ of PPFD. Likewise, *R*_light 0800 h_, *R*_light 1200 h_ and *R*_light 1600 h_ are respiration *R*_light_ measured at 0800, 1200 and 1600 h, respectively.

All gas exchange measurements were taken at a relative humidity of ~30 % by manipulating the amount of air passing through a drying column prior to entering the leaf cuvette. The Li-Cor cuvette block temperature was set to 28 °C for all measurements to account for the influences of leaf temperature on gas exchange variables. In addition, the flow rate was kept at 300 µmol m^−2^ s^−1^.

### Chlorophyll a fluorescence—Soil Plant Analysis Development and JIP-test measurements

Soil Plant Analysis Development (SPAD) values and Chlorophyll *a* fluorescence were measured on the same intact leaves used for the gas exchange measurements and on the same days, also at 0800, 1200 and 1600 h. Five SPAD values were averaged in the same sampled leaf, using the SPAD-502 Chlorophyll Meter (Minolta Co. Ltd, Osaka, Japan). Maximum quantum yield of primary photochemistry (*F*_*v*_*/F*_*m*_) and the performance index [PI—energy cascade processes from the first light absorption event until plastoquinone reduction (Strasser *et al.* 2004)] was measured using a non-modulated fluorimeter model Pocket PEA (Plant Efficiency Analyser, Hansatech, King’s Lynn, Norfolk, UK). The leaves were dark-adapted for *ca.* 30 min using leaf clips (Plant Efficiency Analyser, Hansatech, King’s Lynn, Norfolk, UK) so that all reaction centres of photosystem II (PSII) acquired an ‘open’ status, and heat loss was minimal (Strasser *et al.* 2000).

The JIP-test equations (Strasser and Strasser 1995; Strasser *et al.* 1995; Strasser and Tsimilli-Michael 2001; Strasser *et al.* 2004) were applied to calculate: the effective antenna size of an active reaction centre (RC) (ABS/RC); the maximal trapping rate of PSII (TR_0_/RC); the electron transport in an active RC (ET_0_/RC); the effective dissipation of an active RC (DI_0_/RC); the electron transport probability (ET_0_/TR_0_); the quantum yield of electron transport (ET_0_/ABS); the number of photons absorbed by an excited PSII cross-section (ABS/CS_0_); the maximal trapping rate in a PSII cross-section (TR_0_/CS_0_); the electron transport in a PSII cross-section (ET_0_/CS_0_); the fraction of active reaction centres per excited cross-section of leaf (RC/CS_0_); and the area above the fast fluorescence rise.

### Photosynthetic pigments, dry weight and nitrogen content

At 80 days after transferring plants, leaves, stems, petiole and roots were collected, dried in a forced-air oven at 70 °C for 72 h and weighed to determine the leaf, petiole, stem and root dry weights. The leaf, petiole, stem and root dry weight values were then used to calculate both the biomass allocation (%) and the relation between shoot (leaf dry weight + shoot dry weight + petiole dry weight) and root dry weights (root dry weight/shoot dry weight).

The same leaves used for gas exchange measurements were collected separately in order to determine the photosynthetic pigment contents. Therefore, five leaf discs (each 28.26 mm^2^) were cut into fine strips and placed in a test tube containing 5 mL dimethyl sulfoxide. The test tubes were then incubated at 70 °C for 30 min in the dark. After cooling the extract in the dark, a 3 mL aliquot was analysed spectrophotometrically at 480, 649 and 665 nm (Beckman DU640; Varian, Walnut Creek, CA, USA). Contents of chlorophyll a, chlorophyll b and total carotenoid were calculated according to Wellburn (1994). The remaining parts of the leaves were dried in a forced-air oven at 70 °C for 72 h. Dried leaves were then weighed and ground in a Wiley mill with 20 mesh sieve (Thomas Wiley® Mini-Mill Cutting Mill, Swedesboro, NJ, USA). Leaf powdered aliquots (200 mg) were then solubilized in a solution of sulphuric acid (98 %) in order to determine the level of N according to Kjeldahl semi-micro method (Malavolta *et al.* 1997).

### Statistical analysis

A completely randomized design was used with both genotypes as treatments. Nine replicates were used for both growth and dry weight measurements, totalling 18 plots (plants). Gas exchange, SPAD readings, Chlorophyll *a* fluorescence, photosynthetic pigments and nitrogen content measurements were performed in four replicates, totalling eight plots. In order to compare two groups of normally distributed data (two genotypes), dry weight measurements, gas exchange traits, photosynthetic pigment traits and nitrogen content data were analysed by Student’s unpaired *t*-test at 5 % probability. Although gas exchange measurements were performed throughout the day, comparisons by the *t*-test were made within each measurement time only. For chlorophyll *a* fluorescence data and SPAD readings, a complete randomized design in a split plot scheme was used with 2 genotypes × 3 measurement times × 4 replications, in order to compare both the differences between genotypes within each measurement time, and the differences in these traits among the three measurement times within each genotype. Chlorophyll *a* fluorescence data and SPAD readings were then subjected to analysis of variance tests and the mean pairwise comparisons made using the Tukey HSD test at 5 % probability. Linear regression slopes for WUE (*A*_net_ vs. *E*), iWUE (*A*_net_ vs. *g*_*s*_), Φ_*I*_ (*A*_net_ vs. PPFD), plant height, leaf number, stem diameter and total leaf area were calculated. Statistical analysis was made using the software Sisvar 5.6.

## Results

### Plant growth

**Figure 2.**
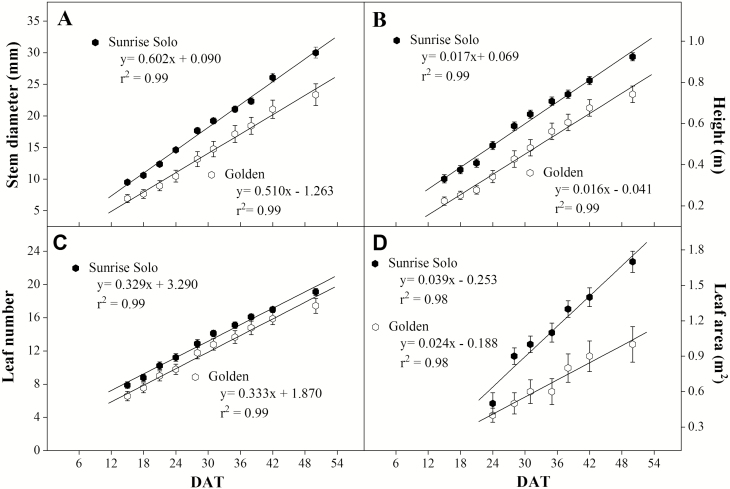
Stem diameter (A), height (B), leaf number (C) and leaf area (D) of two *Carica papaya* genotypes (Golden and Sunrise Solo) along the experiment (DAT—days after transfer to 40 L pots). Each treatment mean represents the average of nine replicates.

**Figure 3.**
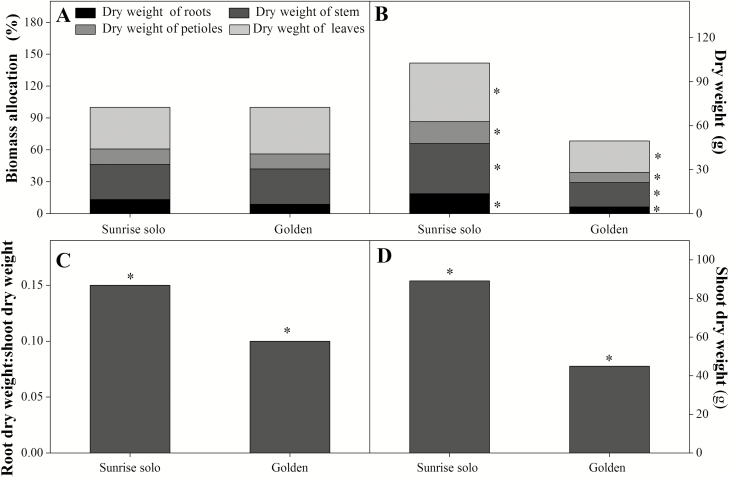
Biomass allocation (A); dry weight (B) of roots, stem, petioles and leaves; root dry weight:shoot dry weight ratio (C); and shoot dry weight (D) of two *Carica papaya* genotypes (Golden and Sunrise Solo). Each column represents the mean of nine replicates. * indicates significant statistical difference by unpaired Student’s *t*-test at 5 % probability.

Sunrise Solo genotype plants had larger stem diameter, height, number of leaves and leaf area throughout the experiment (Fig. 2). Regarding stem diameter, the difference between genotypes increased with time since Sunrise Solo stem diameter growth rate was 14.5 % higher. That led to a difference between genotypes of *ca*. 4 mm at 15 days after transferring plants to *ca*. 8 mm at 54 days after transferring plants (Fig. 2A). Both genotypes had similar plant height and leaf number increment rates (*ca.* 0.02 m day^−1^ and *ca.* 3–4 leaves each 10 days, respectively; Fig. 2B and C). However, despite the lack of genotypic differences in the number of leaves maintained, total leaf area was increasingly different. Sunrise Solo plants had a higher total leaf area growth rate (0.039 m^2^ day^−1^) than the Golden genotype (0.024 m^2^ day^−1^), that is, a 63 % higher rate in leaf area evolution (Fig. 2D). In addition, Sunrise Solo had significantly higher root, leaf, petiole and stem dry weights than Golden (*ca.* 66, 46, 53 and 51 % higher, respectively; Fig. 3B).

Photoassimilates were allocated primarily to the leaves in both genotypes (*ca.* 44 and 39 % in Golden and Sunrise Solo, respectively), as reflected by biomass allocation (Fig. 3A). Less biomass (*ca.* 33 %) was allocated to the roots in Golden than Sunrise Solo (Fig. 3A). Likewise, Golden had significantly lower root dry weight:shoot dry weight ratio (*ca.* 33 %; Fig. 3C) and shoot dry weight (*ca.* 50 %) than Sunrise Solo (Fig. 3D).

### Leaf gas exchanges

**Figure 4.**
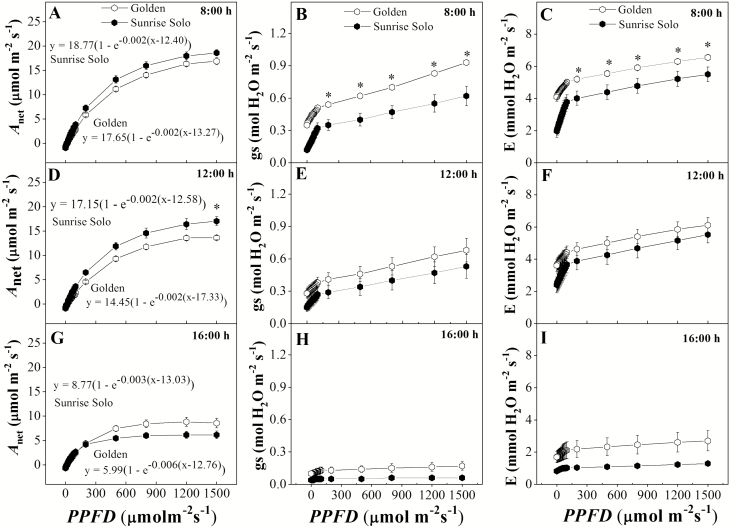
Net photosynthetic rate (*A*_net_—A, D and G), stomatal conductance (*g*_*s*_—B, E and H), transpiration (*E*—C, F and I) of two *Carica papaya* genotypes (Golden and Sunrise Solo) at 0800 (A, B and C), 1200 (D, E and F) and 1600 h (G, H and I). Each point represents the mean of four replicates. Bars represent the standard error. * indicates significant statistical difference for the same PPFD by unpaired Student’s *t*-test at 5 % probability. Golden and Sunrise Solo light compensation points (*I*_*c*_, µmol_PPFD_ m^−2^ s^−1^) were, respectively, 13.27 and 12.40 at 0800 h; 17.33 and 12.58 at 1200 h; and 13.03 and 12.76 at 1600 h. Golden and Sunrise Solo maximum photosynthesis values (*A*_max_, µmol_CO2_ m^−2^ s^−1^) were, respectively, 17.65 and 18.77 at 0800 h; 14.50 and 17.15 at 1200 h; and 8.77 and 5.99 at 1600 h. Golden and Sunrise Solo incident quantum yield values (Φ_*I*_, µmol_CO2_/µmol_PPFD_) were, respectively, 0.032 and 0.041 at 0800 h; 0.026 and 0.037 at 1200 h; and 0.025 and 0.025 at 1600 h.

At 1200 h, only for the highest provided irradiance (1500 μmol m^−2^ s^−1^) the Sunrise Solo genotype had a significantly higher *A*_net_ than Golden (*ca.* 17 vs. *ca.* 14 μmol CO_2_ m^−2^ s^−1^, respectively) (Fig. 4D). Furthermore, no significant differences were observed both 0800 and 1600 h for any of applied irradiances (Fig. 4A and G).

*I*_*c*_ values from Sunrise Solo did not vary throughout the day (*ca*. 12 μmol m^−2^ s^−1^), whereas they changed from *ca.* 13 μmol m^−2^ s^−1^ (0800 and 1600 h) to *ca.* 17 μmol m^−2^ s^−1^ (1200 h) in Golden (see Fig. 4 legend). Furthermore, no significant differences were observed in Φ_*I*_, although mean values were higher at 0800 h in both genotypes (Fig. 4A, D and G).

Golden had significantly higher *g*_*s*_ and *E* values than Sunrise Solo at 0800 h, at PPFD higher than 200 μmol m^−2^ s^−1^ (Fig. 4B and C). Golden maintained somewhat higher values of *g*_*s*_ and *E* throughout the day, although without significant differences (Fig. 4E, F, H and I). Both genotypes had considerably lower *g*_*s*_ and *E* values (*ca*. 16 and 33 %, respectively) at 1600 h in relation to the measurements taken earlier in the day.

As a consequence of the observed variation in *A*_net_, *g*_*s*_ and *E*, Sunrise Solo had a 42 % higher iWUE and 30 % higher WUE than Golden **[see****Supporting Information—Table S1****]**.

**Figure 5.**
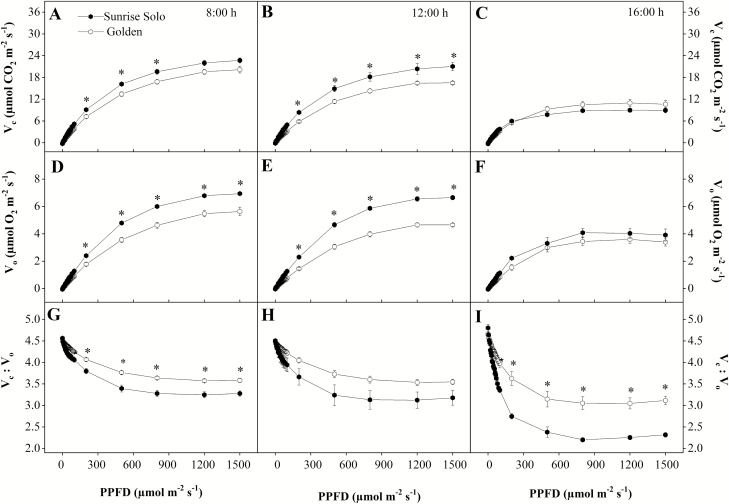
RuBisCO carboxylation (*V*_*c*_—A, B and C) and oxygenation (*V*_*o*_—D, E and F) rates and *V*_*c*_:*V*_*o*_ ratio (G, H and I) of two *Carica papaya* genotypes (Golden and Sunrise Solo) at 0800 (A, B and C), 1200 (D, E and F) and 1600 h (G, H and I). Each point represents the mean of four replicates. Bars represent the standard error. * indicates significant statistical difference by unpaired Student’s *t*-test at 5 % probability.

Following the *A*_net_ pattern, *V*_*c*_ values were higher in Sunrise Solo than in Golden plants at both 0800 and 1200 h, and these differences were statistically significant at PPFD ranging from 200 to 800 μmol m^−2^ s^−1^ (0800 h) and from 200 to 1500 μmol m^−2^ s^−1^ (1200 h) (Fig. 5A and B). These differences were *ca.* 17 % at 0800 h and *ca.* 23 % at 1200 h. Sunrise Solo plants also showed higher *V*_*o*_ values than Golden plants at PPFD values greater than 200 μmol m^−2^ s^−1^, at both 0800 and 1200 h (*ca.* 22 and 32 %, respectively, Fig. 5D and E). No significant differences between genotypes were observed in either *V*_*c*_ or *V*_*o*_ at 1600 h (Fig. 5C and F). Regarding the *V*_*c*_:*V*_*o*_ ratio, Golden showed 8 % higher values than Sunrise Solo at 0800 h and 26 % higher values at 1600 h, when irradiances greater than 200 μmol m^−2^ s^−1^ (Fig. 5G and I).

**Figure 6.**
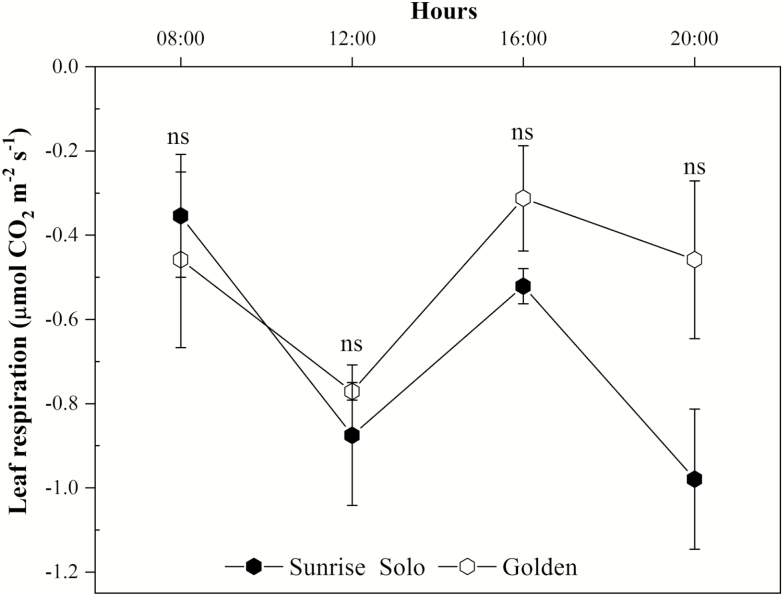
Leaf respiration rates in the light, *R*_light_ (at 0800, 1200 and 1600 h) and in the dark, *R*_dark_ (at 2000 h) of two *Carica papaya* genotypes (Golden and Sunrise Solo). Each point represents the mean of four replicates. Bars represent the standard error. *ns* indicates no statistical difference by unpaired Student’s *t*-test at 5 % probability.

**Figure 7.**
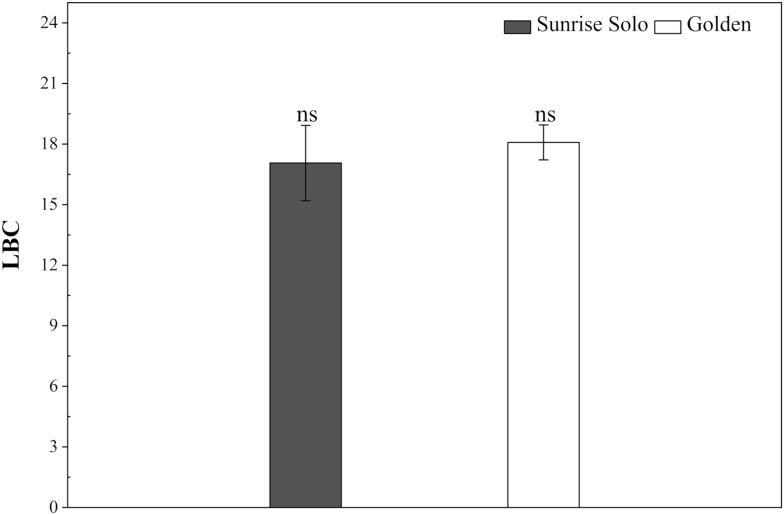
Leaf carbon balance of two *Carica papaya* genotypes (Golden and Sunrise Solo) calculated using net photosynthetic rates (*A*_net_) at 1200 μmol m^−2^ s^−1^ of PPFD. Each column represents the mean of four replicates. Bars represent the standard error. *ns* indicates no statistical difference by unpaired Student’s *t*-test at 5 % probability.

No significant differences were found in *R*_light_ throughout the day, or in *R*_dark_ (leaf respiration rates at 2000 h) (Fig. 6), although Golden plants consistently tended to higher values from 1200 h onwards. Likewise, no significant differences between genotypes were observed in LCB calculated using *A*_net_ values obtained at 1200 μmol m^−2^ s^−1^ (Fig. 7), or 1500 μmol m^−2^ s^−1^**[see****Supporting Information—Fig. S2****]** of PPFD.

### Photosynthetic pigments and nitrogen content, SPAD values and Chlorophyll a fluorescence

**Figure 8.**
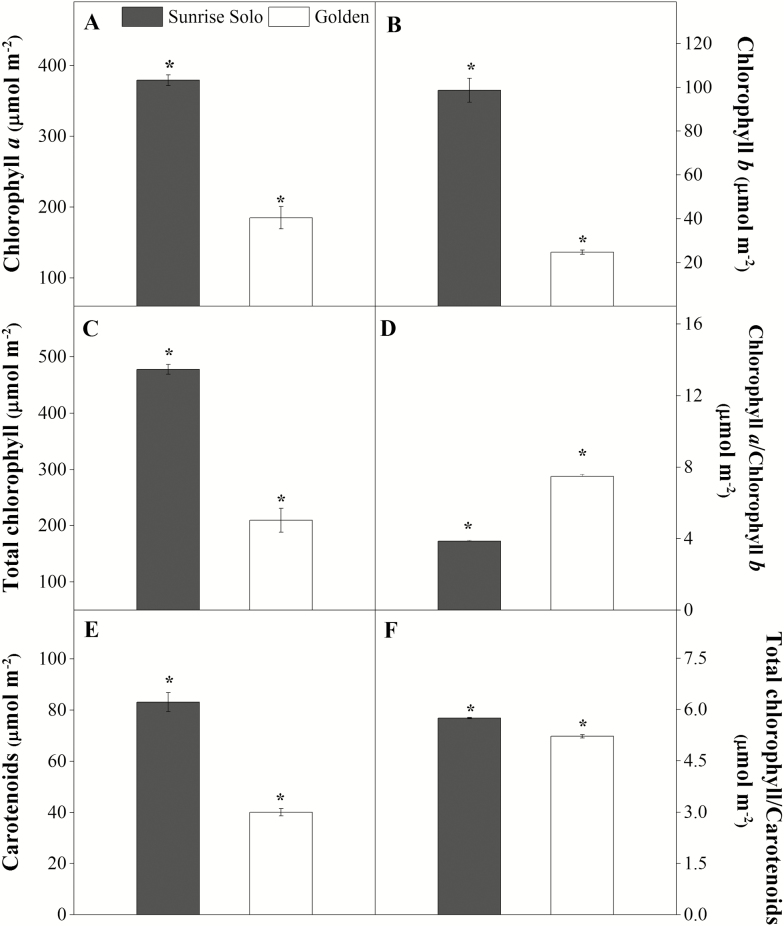
Changes in content of chlorophyll *a* (A), chlorophyll b (B), total chlorophyll (C), chlorophyll *a*:chlorophyll *b* ratio (D), total carotenoids (E) and total chlorophyll:total carotenoids ratio (F) of two *Carica papaya* genotypes (Golden and Sunrise Solo). Each column represents the mean of four replicates. * indicates significant statistical difference by unpaired Student’s *t*-test at 5 % probability within the same day.

**Figure 9.**
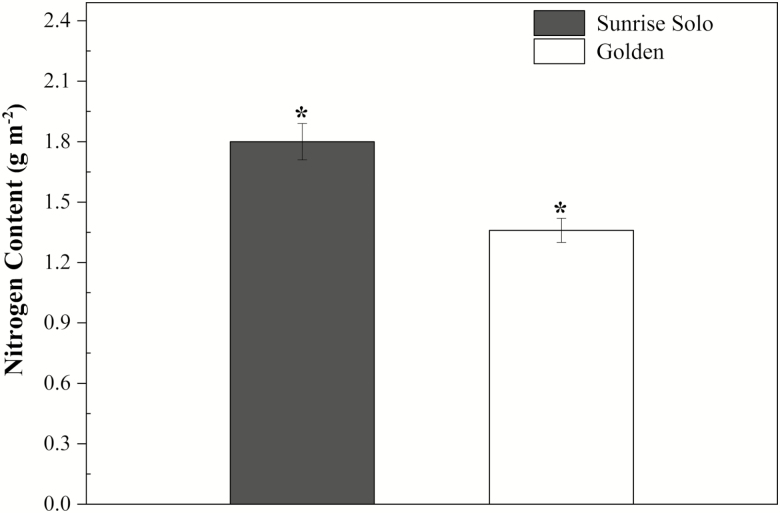
Nitrogen content of two *Carica papaya* genotypes (Golden and Sunrise Solo). Each column represents the mean of four replicates. * indicates significant statistical difference by unpaired Student’s *t*-test at 5 % probability within the same day.

Sunrise Solo showed significantly higher values of chlorophyll *a*, chlorophyll *b*, total chlorophyll, carotenoids and total chlorophyll/carotenoids ratio (51, 75, 56, 52 and 9 %, respectively) (Fig. 8A, B, C, E and F), and lower (*ca.* 48 %) chlorophyll *a*/chlorophyll *b* ratio than Golden (Fig. 8D). In line with the higher content in photosynthetic pigments, greater leaf N content was also observed in Sunrise Solo (*ca.* 11 % greater, Fig. 9). Greater leaf pigments and N content resulted in higher SPAD values in Sunrise Solo than Golden plants [see Fig. 1 and Supporting Information—Table S2].

Overall, both genotypes showed a small variation along the diurnal period for chlorophyll *a* fluorescence parameters **[see****Supporting Information—Table S2****]**. With the exception of the ABS/CS_0_ (both genotypes), ET_0_/RC and RC/CS_0_ (Sunrise Solo), no significant differences were observed among measurements (0800, 1200 and 1600 h).

Regarding the differences between genotypes, no significant differences were found in ABS/RC, DI_0_/RC, RC/CS_0_ and ABS/CS_0_, but Sunrise Solo showed higher values than Golden for TR_0_/RC (0800 h), ET_0_/RC, ET_0_/TR_0_, TR_0_/CS_0_, ET_0_/CS_0_, *F*_*v*_*/F*_*m*_, SPAD and PI throughout the day, as well as larger area at 1200 and 1600 h.

## Discussion

The leaves of Golden showed lower N allocation, photosynthetic pigments (Figs 8 and 9) and, likely, photochemical components than Sunrise Solo leaves as previously reported (Castro *et al.* 2014). This lower investment agreed with the lowered TR_0_/RC, ET_0_/RC, ET_0_/TR_0_, ET_0_/ABS, TR_0_/CS_0_, ET_0_/CS_0_, PI, photochemical efficiency (*F*_*v*_*/F*_*m*_) and SPAD values **[see****Supporting Information—Table S2****]**. Such lower investment in photochemical components will limit the photochemical energy production needed to both carboxylation and oxygenation reactions of RuBisCO (Escalona *et al.* 1997; Hymus *et al.* 2001; Aranjuelo *et al.* 2005; Lambreva *et al.* 2005; Erice *et al.* 2006; Silva *et al.* 2017). In fact, reduced photochemical capacity has been related to reduced chlorophyll contents in other species (Eckardt 2009; Akram and Ashraf 2011). Moreover, in Golden plants, the observed *F*_*v*_*/F*_*m*_ ratio remained <0.69, indicating a reduced photochemical efficiency **[see****Supporting Information—Table S2****]**. Thus, PSII reaction centres might have been impaired, that is, either damaged or inactivated (Baker and Rosenqvist 2004; Zlatev 2009; Rodrigues *et al.* 2016), impartially contributed to blocking electron transfer between the acceptors (Shu *et al.* 2012). In fact, a large portion of leaf N is typically associated with chlorophyll, and specifically to the photosystem I and II cores, and their light-harvesting complexes (Walker *et al.* 2017). Still, these authors observed the maintenance of control rates of canopy photosynthesis in soybean despite a 9 % decrease in leaf N allocation and corresponding decreased chlorophyll. In leaves from Golden plants, the reduction in N content (25 %) and total chlorophyll (−56 %) were even stronger, supporting the proposed impacts on photochemical functions as reflected in the above-mentioned fluorescence parameters and, more moderately, in the observed *A*_net_ (0800 and 1200 h). Additionally, the investment in biochemical components might have been affected, particularly RuBisCO, which is known to be one of the most important N-containing molecules in plant cells (Lawlor 1993; Griffin and Seemann 1996; Zhang *et al.* 2008; Sage 2013). Together these effects could explain the lower *V*_*c*_ and *V*_*o*_ values observed in Golden leaves (Fig. 4). Notably, the impacts in the photo- and biochemical functioning in Golden plants did not significantly affect net CO_2_ uptake through photosynthesis (*A*_net_, Φ_*I*_ and *I*_*c*_; Fig. 4). It is important to point out that although at 1200 h, Sunrise Solo had significantly higher *A*_net_ at 1500 μmol m^−2^ s^−1^ of PPFD (given by the IRGA system) than did Golden (Fig. 4), the maximum ambient PPDF value observed throughout this work was only 882 μmol m^−2^ s^−1^**[see****Supporting Information—Fig. S1****]**. Therefore, the better *A*_net_ response of Sunrise Solo to the high PPFD intensities (~1500 μmol m^−2^ s^−1^) at midday cannot be associated to its improved growth when compared to Golden. However, although there were no significant differences between genotypes, it must be underlined that a consistent tendency for higher *A*_net_ values in Sunrise Solo was observed along the day when compared to Golden, from 200 up to 1500 µmol m^−2^ s^−1^, except for the last daytime measurements. Therefore, although the net photosynthetic rates did not statistically differ between genotypes, we cannot discard the possibility of a cumulative effect, that is, that a sum of a marginal difference can have some impact after a long time period. Such small differences, day after day over a long period, can contribute to some extend to the differences in leaf area investment. The latter can in turn result in greater C-assimilation at whole canopy scale, contributing to the higher yields in Sunrise Solo than in Golden. Specifically, greater *A*_net_ values at midday could contribute to improve Sunrise Solo growth under field conditions in the tropics, where such conditions are observed thought most part of the year.

The higher stomatal conductance of the Golden plants seemed not to affect *A*_net_ (Fig. 4). Nonetheless, the higher *g*_*s*_ and *E*, together with similar *A*_net_ values of Golden compared to Sunrise Solo leaves resulted in reduced iWUE and WUE **[see****Supporting Information—Table S2****]**. Lower WUE confirm earlier results (Torres-Netto *et al.* 2009), and could also be related to observed lower chlorophyll content. In soybean, reduced pigmentation in mutants linked to increase in *g*_*s*_ and reduced iWUE and integrated canopy WUE (Slattery *et al.* 2017). However, since no water limitation was imposed in the present work, we cannot relate the lower growth observed in Golden (Figs 2 and 3) to either iWUE or WUE, or any resulting reduction in plant water availability. Indeed, since *g*_*s*_ means were always higher in Golden plants (Fig. 4), our results show no indications of water stress in this genotype. Previous work published by our research group showed that water-stressed papaya plants dramatically reduced their *g*_*s*_ to values close to 0.06 mol m^−2^ s^−1^ at midday at 1500 μmol m^−2^ s^−1^ of PPFD (Lima *et al.* 2015). Such low *g*_*s*_ values were not observed in our work (Fig. 4E), confirming that our plants did not experience water stress throughout the experiment. Also, abscisic acid concentrations increase in papaya roots under water stress, stimulating root-system growth (Mahouachi *et al.* 2007). Thus, if Golden plants had experienced water stress, we would not have observed the low root dry weight values in Golden (Fig. 3B). Indeed, all pots were fully irrigated until saturation and plants were cultivated in 40 L pots, so that there was no limitation to root-system growth (as visually observed by taking the plants out of the pots at the end of the experiment).

In addition, no significant effects either on leaf respiration (Fig. 5) or on LCB (Fig. 7) were observed between the studied genotypes. Higher LCB values typically represent more carbon available for plant growth (Flexas *et al.* 2006; Escalona *et al.* 2012; Ayub *et al.* 2014). Nonetheless, Sunrise Solo presented higher growth and biomass production than Golden (Figs 2 and 3), despite the absence of large significant differences in *A*_net_, Φ_*I*_, *I*_*c*_, leaf respiration and, consequently, LCB (Figs 4, 6 and 7). Such results show that Sunrise Solo had a higher inherent capacity to convert the available C into biomass than Golden and/or that biomass construction costs are higher in Golden. These findings contradict our initial hypothesis, and thus we suggest that physiological processes other than photosynthesis, leaf respiration and, therefore, LCB are likely to be involved in reduced biomass measured in Golden (Figs 2 and 3). There appears to be an inherent stoichiometry among the various physiological components of the LCB and within the photosynthetic machinery that constrain the conversion of light energy into biomass. Thus, even though both genotypes present similar values of LCB, less biomass is produced in Golden (Figs 2, 3 and 7). Possibly other carbon losses are involved. For example, isoprene emission may be involved and could reduce yield of the Golden genotype. Indeed, in higher plants, almost all of the carbon used to produce isoprene comes directly from photosynthetic intermediates, reducing the amount of C available for plant growth (Loreto and Fineschi 2014).

Although *ca.* 45 % of the dry weight of plants consists of C, biomass production strongly depends on N to synthesize several important molecules (Marschner 1995), such as chlorophylls and RuBisCO, as well as proteins, nucleic acids and various enzymatic cofactors (Griffin and Seemann 1996; Zhang *et al.* 2008; Sage 2013). Since Golden had lower leaf N content than Sunrise Solo (Fig. 9), we suggest that the lower biomass of Golden may be related to either lower N assimilation through the metabolic pathways, which may indirectly affect growth (Figs 2 and 3). Indeed, plants can increase nitrogen assimilation via the photorespiratory pathway, fixing carbon as amino acids in addition to carbohydrates (Busch *et al.* 2018). The higher *V*_*o*_ and lower *V*_*c*_:*V*_*o*_ ratio in Sunrise Solo (Figs 5 and 9) might support higher rates of photorespiration, which in turn, could enable higher rates of N integration in organic compounds (Busch *et al.* 2018). This would justify the observed higher leaf N content, stronger biomass accumulation, and, future, higher yields observed in Sunrise Solo, as compared to Golden (Caliman Agrícola, Linhares, Espiríto Santo, Brazil, pers. comm.). Moreover, the reduction of NO_3_^−^ to NH_4_^+^ associated with photorespiratory nitrogen assimilation is a strong sink of both electrons and reducing power which are photochemically created (Busch *et al.* 2018), and could explain why the improved photochemical capacity of Sunrise Solo **[see****Supporting Information—Table S2****]** did not result in higher CO_2_ uptake rates (*A*_net_) (Fig. 4).

Additionally, nitrate reductase is a key enzyme related to plant growth (e.g. Falxa-Raymond *et al.* 2012), that can be down-regulated under low N availability. We suggest that the lower N content in Golden leaves (Fig. 9) may be the result of having less reducing power available for NO_3_^−^ reduction, and the subsequent impairment of amino acid metabolism and therefore, plant growth. Indeed, Golden leaves had only *ca.* 37 mg N kg^−1^ dry weight (1.3 g m^−2^; Fig. 9), whereas a range of 45–55 g N kg^−1^ dry weight has been considered adequate for papaya leaves (Viégas 1997). The effects of nitrate reductase activity on Golden physiology and growth require further investigations as the effective cultivation of this genotype is likely to be affected by this enzyme activity. Finally, although the reduction in leaf chlorophyll content in Golden has been associated with reduced growth and leaf N content, the lack of change in photosynthetic carbon assimilation may indicate that papaya plants typically produce a ‘luxury’ leaf chlorophyll concentration. This information can be used in breeding programmes with the objective of increasing nitrogen use efficiency (NUE) (*A*_net_/N_leaf_) and avoiding heavy applications of N in papaya fields for higher profitability and better environmental sustainability.

In conclusion, this study provides the first complete picture of LCB in two economically important genotypes of papaya and demonstrates that neither stomatal effects nor reduced photochemical and carboxylation capacities of Golden genotype affected CO_2_ assimilation through photosynthesis. Nonetheless, the accumulation of small differences in photosynthesis, day after day, over a long period, might contribute to some extend to a higher C-budget in Sunrise Solo, higher leaf area and, thus, to higher productivity. Additionally, we consider that physiological processes other than photosynthesis and leaf respiration (LCB) can be as well involved in the lower growth and yield of Golden. One of these aspects could be related to the higher rates of photorespiration observed in Sunrise Solo, which could improve the rate of N assimilation into organic compounds, such as amino acids, thus contributing to the higher biomass production in Sunrise Solo relative to Golden. However, further experiments to evaluate the effects of N metabolism on Golden physiology and growth, as well as measurements of the whole canopy gas exchange and phytohormonal balance are required as these have the potential to affect both growth and yield. In addition, assessments of respiration of trunk and root biomass should be made in both genotypes.

## Sources of Funding

This research was funded by Coordenação de Aperfeiçoamento de Pessoal de Nível Superior - CAPES and Fundação Carlos Chagas de Apoio à Pesquisa do Estado do Rio de Janeiro - FAPERJ (grant E-26/202.323/2017 to W.P.R.), and Conselho Nacional de Desenvolvimento Científico e Tecnológico (CNPq) (fellowship 300996/2016-0 to E.C.), all from Brazil. The authors wish to thank the support Portuguese national funds from Fundação para a Ciência e a Tecnologia through the research units UID/AGR/04129/2013 (LEAF) and UID/GEO/04035/2013 (GeoBioTec).

## Contributions by the Authors

J.S.P., J.R.S., K.F.R., W.P.R., J.A.M.F., K.L.G. and E.C. designed the study. J.S.P., J.R.S., K.F.R., W.P.R., W.P.B., D.P.A. and L.S.F. performed the experiment. J.S.P., J.R.S., K.F.R., W.P.R., J.C.R. and E.C. analysed the data. J.S.P., J.R.S., K.F.R., W.P.R., J.A.M.F., J.C.G., K.L.G., J.C.R. and E.C. wrote the manuscript. All the authors revised the manuscript.

## Conflict of Interest

None declared.

## Supporting Information

The following additional information is available in the online version of this article—

**Figure S1.** Average, minimum and maximum air temperature (A), relative humidity (B), photosynthetic photon flux density (PPFD, from 6:30 am to 5:30 pm) (C) and air vapour pressure deficit (VPD) (D) throughout the experiment. Arrows indicate the days gas exchange variables were measured.

**Figure S2.** Leaf carbon balance (LCB) of two *Carica papaya* genotypes (Golden and Sunrise Solo) calculated using net photosynthetic rates (*A*_net_) at 1500 μmol m^−2^ s^−1^ of photosynthetic photon flux density. Each column represents the mean of four replicates. Bars represent the standard error. *ns* indicates no statistical difference by unpaired Student’s *t*-test at 5 % probability.

**Table S1.** Intrinsic water use efficiency (iWUE) and instantaneous water use efficiency (WUE) of two *Carica papaya* genotypes—Golden and Sunrise Solo (*n* = 12).

**Table S2.** Changes in SPAD readings and in the fluorescence parameters obtained from a JIP-test analysis of two *Carica papaya* genotypes—Golden (G) and Sunrise Solo (SS) (*n* = 4).

Supplementary Information
