# Supplementary material for: Photosynthetic capacity, leaf respiration and growth in two papaya (Carica papaya) genotypes with different leaf chlorophyll concentrations
Source: AoB Plants. 2019 Mar 8;11(2):plz013. doi: 10.1093/aobpla/plz013 (PMC6441136; doi:10.1093/aobpla/plz013)
Supplement: Supplementary Information [file plz013_suppl_supplementary_information.pdf]

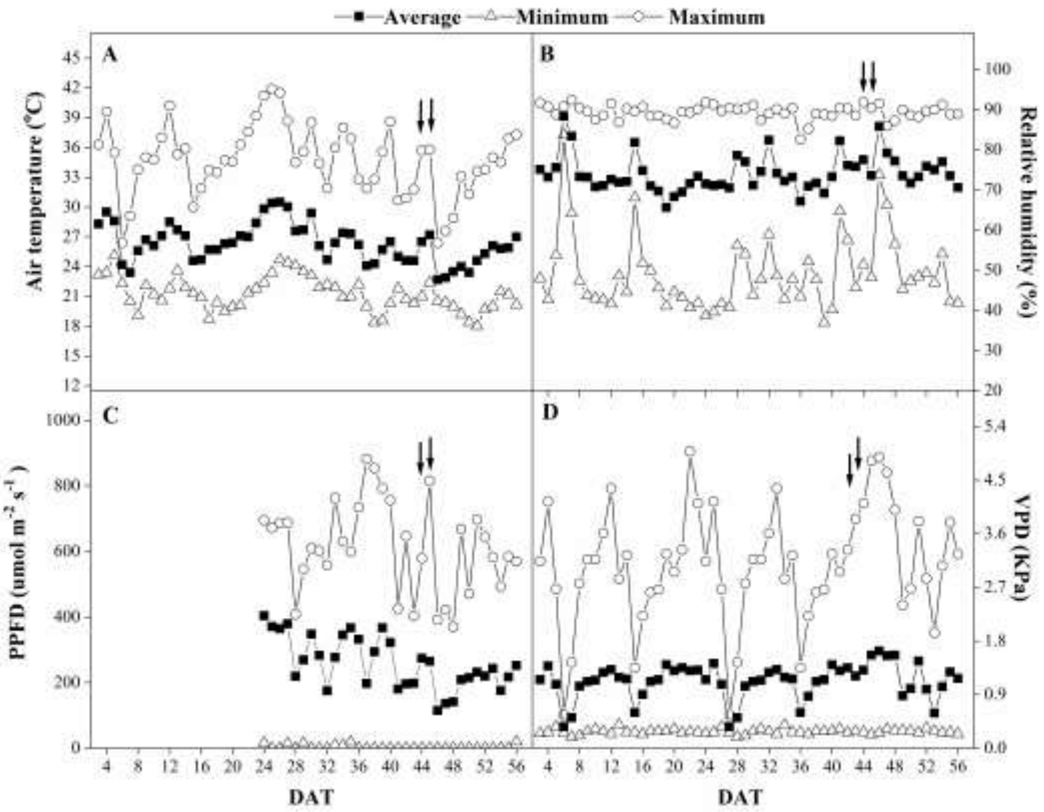

Average
Minimum
Maximum
A
B
C
D
Air temperature (°C)
Relative humidity (%)
PPFD (umol m-2 s-1)
VPD (KPa)
DAT
DAT

Figure S1

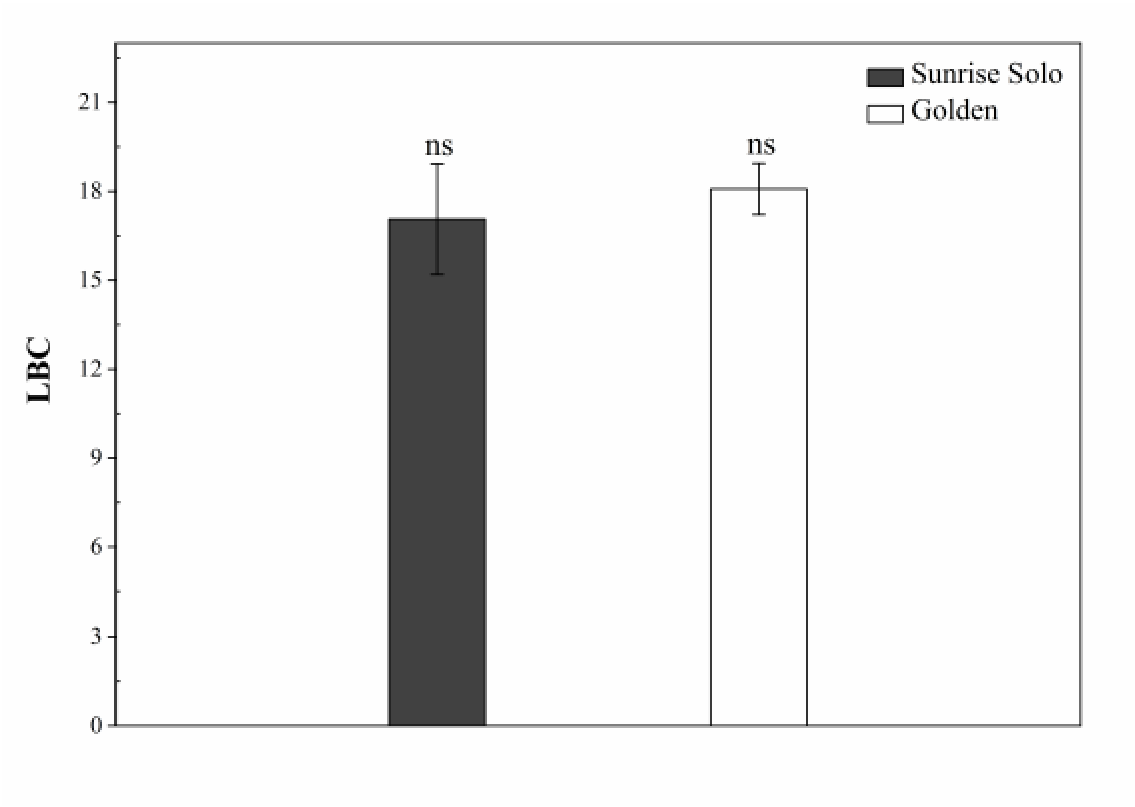

Figure S2

Supplementary tables

Supplementary Table S1. Intrinsic Water Use Efficiency (*iWUE*) and instantaneous Water Use Efficiency (*WUE*) of two *Carica papaya* genotypes – Golden and Sunrise Solo – (n = 12).

Genotypes	*iWUE*	R^2	*WUE*	R^2
Sunrise Solo	y = 18.2540x + 5.3258	0.62	y = 2.3535x + 2.7905	0.70
Golden	y = 10.6837x + 5.2637	0.56	y = 1.6241x + 2.8582	0.50

Supplementary Table S2. Changes in SPAD readings and in the fluorescence parameters obtained from a JIP Test analysis of two *Carica papaya* genotypes - Golden (G) and Sunrise Solo (SS) (n=4).

Genotype	08:00	12:00	16:00
ABS/RC			
SS	1.40 ± 0.05 Aa	1.57 ± 0.06 Aa	1.29 ± 0.05 Aa
G	1.43 ± 0.19 Aa	1.68 ± 0.16 Aa	1.41 ± 0.11 Aa
TR_o/RC			
SS	1.07 ± 0.02 Aa	1.10 ± 0.03 Aa	0.97 ± 0.02 Aa
G	0.94 ± 0.06 Ba	1.01 ± 0.04 Aa	0.93 ± 0.04 Aa
ET_o/RC			
SS	0.65 ± 0.01 Aa	0.68 ± 0.02 Aa	0.57 ± 0.01 Ab
G	0.38 ± 0.03 Ba	0.32 ± 0.03 Ba	0.31 ± 0.03 Ba
DI_o/RC			
SS	0.33 ± 0.02 Aa	0.47 ± 0.04 Aa	0.32 ± 0.03 Aa
G	0.48 ± 0.03 Aa	0.67 ± 0.12 Aa	0.48 ± 0.07 Aa
ET_o/TR_o			
SS	0.60 ± 0.01 Aa	0.61 ± 0.01 Aa	0.59 ± 0.01 Aa
G	0.41 ± 0.55 Ba	0.32 ± 0.30 Ba	0.34 ± 1.58 Ba
ET_o/ABS			
SS	0.46 ± 0.01 Aa	0.43 ± 0.01 Aa	0.44 ± 0.02 Aa
G	0.28 ± 0.78 Ba	0.20 ± 0.20 Ba	0.22 ± 0.35 Ba
ABS/CS_o			
SS	5900 ± 250 Ab	7107 ± 249 Aa	6556 ± 338 Aab
G	5800 ± 122 Ab	6785 ± 387 Aa	6359 ± 189 Aab
TR_o/CS_o			
SS	4504 ± 149 Aa	4991 ± 129 Aa	4920 ± 175 Aa
G	3931 ± 172 Ba	4132 ± 263 Ba	4204 ± 42 Ba
ET_o/CS_o			
SS	2730 ± 69 Aa	3088 ± 60 Aa	2898 ± 67 Aa
G	1641 ± 212 Ba	1340 ± 175 Ba	1439 ±149 Ba
RC/CS_o			
SS	4198 ± 86 Ab	4520 ± 187 Aab	5061 ± 75 Aa
G	4233 ± 373 Aa	4115 ± 328 Aa	4534 ± 183 Aa
Área			
SS	4.50x105 ± 0.23x105 Aa	5.86x105 ± 1.13x105 Aa	5.46x105 ± 0.41x105 Aa
G	2.99x105 ± 0.61x105 Aa	2.13x105 ± 0.66x105 Ba	2.63x105 ± 0.33x105 Ba
F_v/F_m			
SS	0.78 ± 0.01 Aa	0.72 ± 0.02 Aa	0.76 ± 0.02 Aa
G	0.69 ± 0.03 Ba	0.63 ± 0.03 Ba	0.68 ± 0.02 Ba
PI			
SS	3.42 ± 0.39 Aa	2.34 ± 0.33 Aa	3.41 ± 0.64 Aa
G	1.28 ± 0.36 Ba	0.55 ± 0.21 Ba	0.79 ± 0.20 Ba
SPAD			
SS	53.00 ± 0.7 Aa	53.80 ± 0.9 Aa	53.30 ± 0.9 Aa
G	34.50 ± 0.8 Ba	35.70 ± 0.6 Ba	33.80 ± 0.8 Ba

Each value represents the mean ± SE (n=4); different letters indicate significant differences among measurement times within each genotype (a, bB) or between genotypes within each measurement time (A, B), by Tukey's test at 5% probability.
